# Liver-Targeted Combination Therapy Basing on Glycyrrhizic Acid-Modified DSPE-PEG-PEI Nanoparticles for Co-delivery of Doxorubicin and Bcl-2 siRNA

**DOI:** 10.3389/fphar.2019.00004

**Published:** 2019-01-22

**Authors:** Guixiang Tian, Ruiyan Pan, Bo Zhang, Meihua Qu, Bo Lian, Hong Jiang, Zhiqin Gao, Jingliang Wu

**Affiliations:** ^1^School of Bioscience and Technology, Weifang Medical University, Weifang, China; ^2^School of Pharmacy, Weifang Medical University, Weifang, China

**Keywords:** combination therapy, nanoparticles, delivery, liver cancer, glycyrrhizic acid

## Abstract

Combination therapy based on nano-sized drug delivery system has been developed as a promising strategy by combining two or more anti-tumor mechanisms. Here, we prepared liver-targeted nanoparticles (GH-DPP) composed of 1,2-distearoyl-sn-glycero-3-phosphoethanolamine-polyethylene glycol-polyetherimide (DSPE-PEG-PEI) with Glycyrrhetinic acid-modified hyaluronic acid (GA-HA) for co-delivery of doxorubicin (DOX) and Bcl-2 siRNA. Particles size, zeta potential and morphology were determined for the drug-loaded GH-DPP nanoparticles (siRNA/DOX/GH-DPP). Cellular uptake and *in vitro* cytotoxicity were analyzed against HepG2 cells. *In vivo* bio-distribution and anti-tumor therapeutic effects of siRNA/DOX/GH-DPP were evaluated in H22-bearing mice. The results showed that siRNA/DOX/GH-DPP nanoparticles were nearly spherical and showed dose-dependent cytotoxicity against HepG2 cells. Compared to Glycyrrhetinic acid-free co-delivery system (siRNA/DOX/DPP) and GH-DPP nanoparticles for delivery of DOX or Bcl-2 siRNA alone, siRNA/DOX/GH-DPP nanoparticles could induce more cellular apoptosis, and showed higher anti-tumor effect. Herein GH-DPP nanoparticles could simultaneously deliver both chemotherapy drugs and siRNA into the tumor region, exhibiting great potential in anti-tumor therapy.

## Introduction

Liver cancer is one of prevalent cancers with high mortality rate around the world, and traditional chemotherapy is one effective approach used in anti-cancer therapy (Gravitz, 2014; Sia et al., 2017). However, many chemotherapeutic agents, such as DOX and paclitaxel, have many clinical limitations owing to severe system toxicity, non-specific targeting, and the development of multidrug resistance (MDR) (Zahreddine and Borden, 2013).

To improve selectivity toward liver cancer cells, an effective strategy is to design nano-sized carrier to realize liver-targeted delivery (Shamay et al., 2018). Recently, nanoparticles have been proved to have the advantages in drug delivery with low system toxicity (Wei et al., 2015; Zeng et al., 2017; Arms et al., 2018). Many nano-sized drug delivery systems, such as natural and synthetic polymer nanoparticles, metal nanoparticles, and polymer-drug conjugates, have been investigated for delivery of anti-tumor drugs (Ekladious et al., 2018; Liu et al., 2018; Maeki et al., 2018). The nano-vehicles basing on phosphoethanolamine-polyethylene glycol polymers (PEG-PE) represent a promising nanoparticles delivery system owing to biocompatibility, prolonged circulation, and accumulation in tumors by the enhanced permeability and retention (EPR) effect (Perche et al., 2012; Kohay et al., 2017). In the past decade, many efforts have been made to prepare liver-targeting nano-carriers, which were modified by sugars, antibodies, and other ligands (Singh et al., 2016; Zhu et al., 2016; Yan et al., 2017; Wu J. et al., 2018). Glycyrrhetinic acid (GA), a metabolite of glycyrrhizin, has attracted growing interest in anti-hepatoma therapy (Wu J. et al., 2018). It has been reported that GA-modified nano-carriers could significantly improve liver-targeting efficiency and inhibit liver cancer development.

Moreover, development of MDR in cancer cells was a major cause of the failure in clinical chemotherapy. Bcl-2, an anti-apoptosis protein, is distributed on the endoplasmic reticulum, the outer membrane of nuclear and mitochondrion. Up-regulation of Bcl-2 expression was one of the mechanisms responsible for MDR, leading to the activation of anti-apoptotic pathways (Yin et al., 2014). The Bcl-2 siRNA, an antisense oligonucleotide sequence of Bcl-2, could silence the expression of Bcl-2 gene, resulting in cell apoptosis of liver cancer (Sun et al., 2018).

To overcome the limitations of traditional chemotherapy in clinical antitumor therapy, combination drug strategy has been applied as a novel anti-tumor therapy. It is based on co-delivery nanoparticles system for combination of chemotherapeutics with other treatment approaches like RNAi (Zuckerman and Davis, 2015). The nanoparticles can simultaneously co-deliver two or more drugs to tumor region and thus improve the cancer therapeutic effect by synergistic/combined therapy effect, and reverse the multi-drug resistance (MDR) (Zhang et al., 2016; Sun et al., 2018).

In previous study, we have prepared GA-modified hyaluronic acid micelles for DOX delivery (Wu et al., 2016). Hyaluronic acid (HA), a negatively charged polysaccharide, is present in the extracellular matrix and synovial fluids (Knopf-Marques et al., 2016). It can cover on the shell of positive nano-carriers, such as PEI-PE, chitosan, dendrimer, to decrease the uptake rate by reticuloendothelial systems (Nguyen and Alsberg, 2014; Zhao et al., 2016; Wickens et al., 2017; Parmar et al., 2018).

**FIGURE 1:**
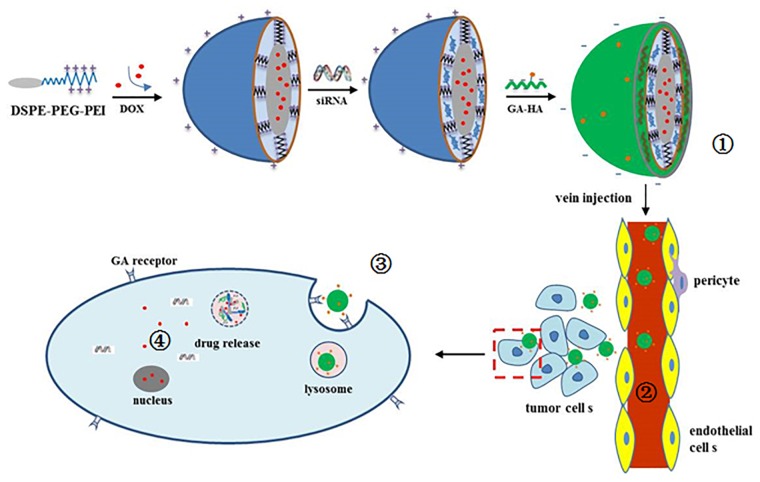
Schematic illustration of 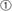 preparation of siRNA/DOX/GH-DPP nanoparticles, 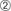 liver-targeted drug delivery via blood cycle, 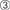 cellular uptake, and 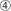 pH-triggered release of Bcl-2 siRNA and DOX.

In this study, DSPE-PEG-PEI and GA-HA conjugates were synthesized, and GH-DPP nanoparticles were prepared for co-delivery of DOX and Bcl-2 siRNA (Figure 1). The characteristics of the drug-loaded nanoparticles were investigated using dynamic light scattering, transmission electron microscopy (TEM) and UV-Vis spectrophotometer. The *in vitro* cytotoxicity and cellular uptake of siRNA/DOX/GH-DPP were investigated against HepG2 cells. And the *in vivo* bio-distribution and anti-tumor effect were explored.

## Materials and Methods

### Materials and Cell Lines

Branched poly(ethyleneimine) (PEI, Mw 1.8 kDa) was purchased from Sigma Aldrich (United States). DOX was purchased from Dalian Meilun Biology Technology Co., Ltd., (Dalian, China). 4-(4,6-dimethoxy-1,3,5-triazin-2-yl)-4-methylmorpholinium chloride (DMT–MM) were purchased from Shanghai Medpep Co., Ltd., (Shanghai, China) 1,2-distearoyl-sn-glycero-3-phosphoethanolamine-N-[succinimidyl (polyethylene glycol) -2000] (DSPE-PEG-NHS) was purchased from Xi’an Rixi Technology Co., Ltd., (Xi’an, China). Bcl-2 siRNA and FITC-labeled siRNA were purchased from Guangzhou RiboBio Co., Ltd., (Guangzhou, China). 3-(4, 5-dimethylthiazol-2-yl)-2, 5-diphenyltetrazolium bromide (MTT) was purchased from Sigma Aldrich (United States). Fetal bovine serum and RPMI-1640 medium (RPMI) were purchased from Beijing Solarbio Co., Ltd., (Beijing, China). All other reagents were of commercial special grade and used without further purification.

Human hepatic cell line (HepG2), human lung adenocarcinoma cell line (A549) and murine HCC cells (H22) were obtained from the China Center for Type Culture Collection (Wuhan, China). Female BALB/c mice (weight: 18 ± 2 g) were supplied by the Experimental Animal Center of Weifang Medical University (Weifang, China), and approved by the WFMU Animal Research Ethics Committee.

### Synthesis of HA-GA and DSPE-PEG-PEI Conjugates

GA-HA conjugate (GH) was synthesized using HA as a hydrophilic segment and GA as a hydrophobic segment (Wu et al., 2016). In brief, GA–NH_2_ was obtained by adding ethylene diamine to the GA solution in the presence of DMT-MM. And the GA–HA conjugate was synthesized by the chemical modification of GA–NH_2_ to HA chain.

**FIGURE 2:**
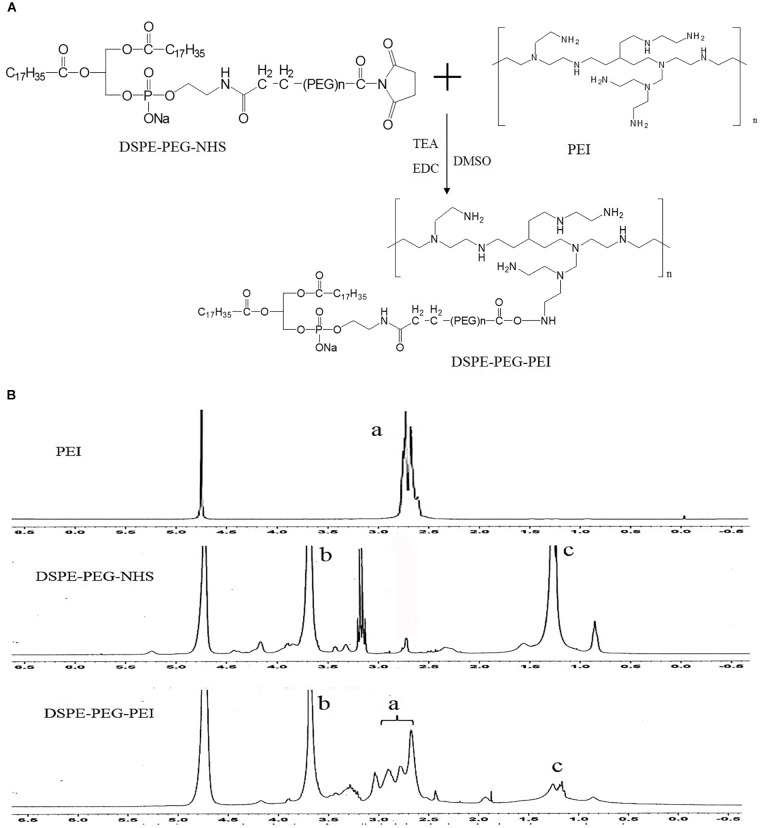
Synthesis of DSPE-PEG-PEI conjugate. **(A)** Synthetic route of DSPE-PEG-PEI conjugate. **(B)** ^1^H-NMR spectra of PEI, DSPE-PEG-NHS and DSPE-PEG-PEI (a: peaks of PEI; b and c: peaks of DSPE-PEG-NHS).

Syntheses of DSPE-PEG-PEI (DPP) were conducted in one steps as shown in Figure 2. Briefly, PEI was dissolved in DMSO (10 mL) in a 25 mL glass flask, and then functional DSPE-PEG-NHS was added into the reaction solution under stirring. The reaction solution was stirred for 24 h at room temperature. The product was purified by dialysis against distilled water (MWCO 8000-14000 Da), lyophilized, and the chemical structure was confirmed by ^1^H NMR (in D_2_O, 300 MHz).

### Preparation and Characteristics of Drug-Loaded GH-DPP Nanoparticles

siRNA/DOX/GH-DPP nanoparticles were prepared by three steps. Firstly, DOX was loaded into the core of DPP nanoparticles via a dialysis method. In brief, DOX • HCl was stirred with triethylamine (1.3-fold molar quantity of DOX) in DMF, and the DPP conjugates were dispersed in formamide. Then the DOX solution was added slowly to the DPP solution, followed by stirring overnight. The mixed system was dialyzed against deionized water. The solution in the dialysis bag was freeze-dried to obtain DOX-loaded DPP nanoparticles (DOX/DPP). Secondly, the DPP nanoparticles for co-delivery of DNA and siRNA were prepared by electrovalent interaction. The sequences of Bcl-2 siRNA were as follows: (sense) 5′ – GUACAUCCAUUAUAAGCUGUCdTdT-3′, (anti-sense) 5′ – GACAGCUUAUAAUGGAUGUACdTdT-3′. DOX/DPP nanoparticles were incubated with Bcl-2 siRNA in deionized water. In order to obtain the proper mass ratio of DPP to siRNA, the same amount of siRNA was incubated with different concentrations of DOX/DPP nanopaticles solutions for 1 h. The mass ratios of DOX/DPP to siRNA was set as 100:512, 100:256, 100:128, 100:64, 100:32, 100:16, and 100:8, respectively. The binding ability of DOX/DPP and siRNA was investigated by agarose gel retardation assay, followed by electrophoretic mobility shift assay via a UV gel imaging system. The proper mass ratio of DOX/DPP to siRNA was selected for preparation of siRNA/DOX/DPP nanoparticles. Thirdly, GA-HA conjugate was mixed with siRNA/DOX/DPP nanoparticles to prepare siRNA/DOX/GH-DPP by stirring slowly for 1 h. Then drug-loaded nanoparticles were freeze-dried, and the lyophilized power was stored at 4°C. The GH-DPP nanoparticles for delivery of DOX or siRNA alone were prepared as control.

The particle size and ζ potential of siRNA/DOX/GH-DPP nanoparticles were measured using a dynamic laser scattering method with a wavelength of 633 nm at 25°C. The detection angle was set to 90°. The polydispersingindex (PdI) was used to evaluate the size distribution. The concentration of siRNA/DOX/GH-DPP nanoparticles was kept 1 mg/mL, and all measurements were performed in triplicate. The morphology of siRNA/DOX/GH-DPP nanoparticles was observed by electron microscopy. One drop of drug-loaded nanoparticles solution were placed on a copper grid, and dried at room temperature. The sample was examined using a transmission electron microscope.

To evaluate the loading efficiency (LE) and encapsulation efficiency (EE) of GH/DPP nanoparticles, siRNA/DOX/GH-DPP nanoparticles were dissolved in formamide by gently heating, and measured using UV–Vis spectrophotometer at 480 nm. The concentration of DOX in the GH/DPP micelles was obtained using the standard curve. Then LE and EE were calculated using the following equation (1) and (2):

(1) LE(%)=Ws/Wt × 100%

(2) EE(%)=Ws/Wa × 100%

Ws = the amount of DOX measured in the GH/DPP nanoparticles; Wt = the total weight of siRNA/DOX/GH-DPP nanoparticles; and Wa = the initial amount of the DOX•HCl added.

### *In vitro* Drug Release From GH-DPP Nanoparticles

The release of DOX and siRNA from GH-DPP nanoparticles was investigated in PBS buffer (pH 7.4 and 5.0) (Wang et al., 2016). 1 mg/mL siRNA/DOX/GH-DPP nanoparticles was dispersed in 5 mL PBS, and the solution was placed in a dialysis bag (MWCO of 1000 and 20000 for DOX and siRNA, respectively). Then, the dialysis bag was placed in 20 mL of PBS buffer at 37°C under a shaking speed of 100 rpm. At predetermined time intervals, 1 mL of release media was taken out and 1 mL of fresh PBS buffer was added. The DOX and siRNA content was tested by UV-Vis spectroscopyat 480 and 260 nm, respectively. The release of DOX and siRNA was calculated by standard curve. The test was performed in triplicate.

### Cytotoxicity Assay of siRNA/DOX/GH-DPP Nanoparticles

The cytotoxicity of blank DPP and GH-DPP nanoparticles against HepG2 and A549 cells was evaluated by MTT assay. Briefly, the tumor cells were seeded in 96-well plates (1 × 10^4^ cells/well) and incubated for 48 h. Then, the cells were co-cultured with different concentrations (1, 10, 20, 50, and 100 μg/ml) of DPP or GH-DPP nanoparticles, respectively. After 48 h, 20 μL of MTT reagents (5 mg/mL) was added for another 4 h incubation at 37°C. The media were replaced with 200 μL of DMSO. The absorbance at 490 nm was measured using a Bio-Rad Microplate Reader (Model 680, Richmond, VA, United States).

The cytotoxicity of siRNA/DOX/GH-DPP nanoparticles was evaluated by MTT assay against HepG2 and A549 cells. The cells were incubated with the culture media containing free DOX, DOX/GH-DPP, siRNA/GH-DPP, siRNA/DOX/DPP and siRNA/DOX/GH-DPP nanoparticles at different DOX concentrations (0.01, 0.1, 0.5, 1, 2, and 5 μg/mL), respectively. The cytotoxicity of drug formulations was shown as a cell viability percentage with respect to the untreated tumor cells. All the experiments were repeated thrice.

### Cellular Uptake Analysis

Cellular uptake of DOX and FITC-labeled siRNA was monitored by fluorescent microscopy (BX40, Olympus, Japan). HepG2 cells were seeded in a 12-well plate at a density of 1 × 10^5^ cells/well at 37°C. After the cells reached 75% confluence, the media were replaced with fresh media containing free DOX and siRNA, siRNA/DOX/DPP, siRNA/DOX/GH-DPP nanoparticles, respectively. After 4 h, the cells were washed three times by cold PBS, and fixed with 4% paraformaldehyde solution. The intracellular localization of DOX was visualized by fluorescence microscope.

### Western Blotting Analysis

Suppression of the BCL-2 protein was determined by Western blot using bicin-choninic acid protein assay kit (BCA, Invitrogen, United States). Sample proteins (30 μg) was subjected to electrophoresis in 10% sodium dodecyl sulfate polacrylamine gel. And the protein was transferred to polyvinylidene difluoride membranes, followed by incubation with non-fat milk for 1 h, and with antibody against BCL-2 and β-action (1:1000 dilution) for 12 h at 4°C. The membranes were washed thrice in TBST, and incubated with HRP conjugated goat anti-rabbit IgG (1:5000, Santa Cruz Biotech., United States) for 1 h. the complexes were visualized using chemiluminescence kit (KeyGEN, China).

### *In vivo* Near-Infrared Fluorescence Imaging (NIFI)

*In vivo* biodistribution of the drug-loaded GH-DPP nanoparticles was monitored via near-infrared fluorescence imaging system. Preparation of DiR loaded GH-DPP nanoparticles was as followed: GH-DPP and DiR were dis-solved in methanol, and the solution was dripped to deionized water by a micro-syringe pump under magnetic stirring. The mixture system was dialyzed against deionized water for 48 h. The final concentration of DiR for tail vein injection was 40 μg/mL. The tumor-bearing mice model was established by subcutaneous inoculation of H22 cells in the flank of BALB/c female mice. When the volume of the tumor grew to approximately 100 mm^3^, the mice were randomly divided into three groups. DiR was used as a fluorescence agent. DiR-loaded DPP and DiR-loaded GH-DPP nanoparticles were prepared, respectively. Free DiR, DiR-loaded DPP and DiR-loaded GH-DPP nanoparticles were administrated by intravenous injection. The *in vivo* near-infrared fluorescence imaging was performed at pre-determined times (2, 6, 12, and 24 h), using the Xenogen IVIS Spectrum from Caliper Life Sciences (Ex was 745 nm, Em was 835 nm).

### Anti-tumor Effect Analysis

The therapeutic effects of drug-loaded GH-DPP nanoparticles were investigated through evaluation of their anti-tumor effects using H22 tumor-bearing mice as model. When the tumor size reached about 100 mm^3^, H22-bearing mice was randomly divided into sever groups (five mice per group). The mice were administrated by physiological saline (control), blank GH-DPP nanoparticles, free DOX•HCl, siRNA/GH-DPP, DOX/GH-DPP, siRNA/DOX/DPP, and siRNA/DOX/GH-DPP nanoparticles, respectively. Drug treatment was set at a dose of 5 mg DOX/kg body weight every other day. The body weight and tumor volume was measured every day. Finally, all of the mice were sacrificed, and the tumors were harvested. The tumor volume was calculated by follow equation:

$$\mathrm{Vt}\;=\;d^{2}\;\times \;L/2$$

L is the longest diameter of tumor; d is the shortest diameter of tumor; and Vt is the tumor volume.

### Statistical Analysis

All results are presented as mean ± S.D., *n* = 3 parallel samples. The data were analyzed by Student’s *t*-test for comparison of two groups. A *p*-value less than 0.05 was considered to be significant.

### Synthesis of DSPE-PEG-PEI Conjugates

Bi-functional DSPE-PEG-NHS was used to conjugate with PEI via the primary amine reactive NHS ester moiety at weakly basic pH, thus avoiding the conjugation and crosslinking of the maleimide groups to the amine functions of PEI, which occurs at higher pH (pH > 8). The structure of DSPE-PEG-NHS, PEI and resulting DSPE-PEG-PEI copolymer were verified by ^1^H NMR. The peaks of PEG (3.6 ppm, -CH_2_O-), DSPE (1.0–1.5 ppm, -CH_2_-) and PEI (2.5–3.0 ppm, CH_2_-N) were confirmed. The^1^H-NMR spectrum of DSPE-PEG-PEI in D_2_O exhibited characteristic peaks at 2.5–3.0 ppm (peaks of PEI), 3.6 ppm (peaks of PEG) and 1.0–1.5 ppm (peaks of DSPE), indicating that PEI was successfully introduced to the DSPE-PEG-NHS molecular.

### Preparation and Physicochemical Characteristics of Drug-Loaded Nanoparticles

**FIGURE 3:**
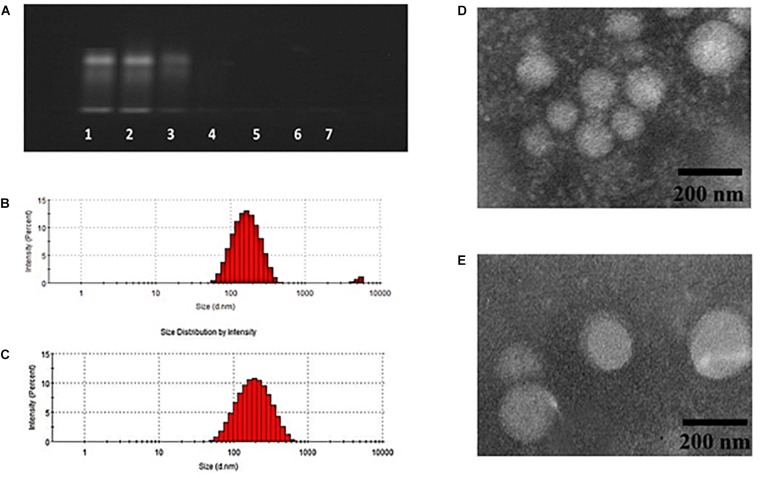
Characteristics of siRNA/DOX-loaded GH-DPP nanoparticles. **(A)** The siRNA retardation assay of GH-DPP at the mass ratio of DPP to siRNA from 100:256 to 100:4 (1, 100:128; 2, 100:64; 3, 100:32; 4, 100:16; 5, 100:8; 6, 100:4). **(B–C)** Particle size distribution of siRNA/DOX/ DPP and siRNA/DOX/GH-DPP nanoparticles. **(D–E)** TEM image of siRNA/DOX/ DPP and siRNA/DOX/GH-DPP nanoparticles.

Doxorubicin and Bcl-2 siRNA were loaded in DPP or GH/DPP copolymers, named as siRNA/DOX/DPP and siRNA/DOX/GH-DPP, respectively. The characterization of DOX-loaded nanoparticles was shown in Table 1. The average particles size of siRNA/DOX/GH-DPP was bigger than that of siRNA/DOX/DPP, while the ζ potential was lower in siRNA/DOX/GH-DPP. The result was due to the coverage of GA-HA conjugate, resulting in bigger particles size and less ζ potential. LE and EE of DOX in siRNA/DOX/GH-DPP nanoparticles were measured by UV spectrophotometer. When the feed ratio of DOX to DPP was 10%, the EE and LE of DOX was 86.1 and 8.02%, respectively. To obtain the co-delivery system of DOX and siRNA, DOX/DPP and siRNA with different mass ratio were mixed and tested by gel retardation assay. Figure 3A showed that the fraction of free DNA disappeared at 100:16, suggesting that DOX/DPP could condense DNA efficaciously when the mass ratio of DPP to siRNA was over 100:16. The siRNA/DOX/DPP and siRNA/DOX/GH-DPP nanoparticles were well-separated with a rather narrow size distribution (Figures 3B,C). As shown in Figures 3D,E, the co-delivery system exhibited sphere in shape. Stability studies showed that drug-loaded GH-DPP nanoparticles were more stable than drug-loaded DPP nanoparticles under physiological conditions (Supplementary Figure S1).

**Table 1:** The particle size, polydispersity index (PDI) and zeta potential of siRNA/DOX/DPP and siRNA/DOX/GH-DPP (*n* = 3).

	Size (nm)	PDI	Zeta (mV)	EE^b^(%)	DL^b^(%)
siRNA/DOX/DPP	157.2 ± 5.7	0.272 ± 0.05	12.75 ± 2.19	87.4 ± 2.7	8.32 ± 1.4
siRNA/DOX/GH-DPP	185.4 ± 6.4^a^	0.294 ± 0.04	-2.64 ± 1.73^a^	86.1 ± 3.1	8.02 ± 1.6


*^*a*^*P* < 0.05 siRNA/DOX/DPP vs siRNA/DOX/GH-DPP. ^*b*^DOX loadeding.*

### DOX and siRNA Release From siRNA/DOX/GH-DPP Nanoparticles

**FIGURE 4:**
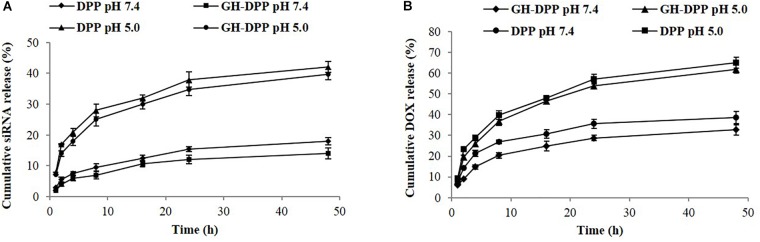
Release profile of siRNA and DOX-loaded nanoparticles. **(A)** siRNA release from GH-DPP or DPP nanoparticles in pH 7.4 or 5.0, respectively. **(B)** DOX release from GH-DPP or DPP nanoparticles in pH 7.4 or 5.0, respectively.

The release of DOX and siRNA from siRNA/DOX/GH-DPP or siRNA/DOX/DPP nanoparticles was conducted in pH 7.4 and pH 5.0. The siRNA and DOX released from GA-DPP or DPP were time-dependent (Figure 4). Both GH-DPP and DPP nanoparticles showed a rapid release at pH 5.0. By contrast, the drug release was slower at pH 7.4. The possible explanation is that the electrostatic interaction between positive segments (PEI) and negative segments (siRNA, GA-HA) is weak at lower pH value, leading to rapid release of the drug from the nano-carriers (Sun et al., 2018). Compared to siRNA/DOX/GH-DPP nanoparticles, the siRNA/DOX/DPP released more drugs at the same time. This may due to the fact the coverage layer (GA-HA) could delay the release of DOX from GH-DPP nanoparticles (Manna et al., 2010).

### *In vitro* Cytotoxicity of siRNA/DOX/GH-DPP Nanoparticls

**FIGURE 5:**
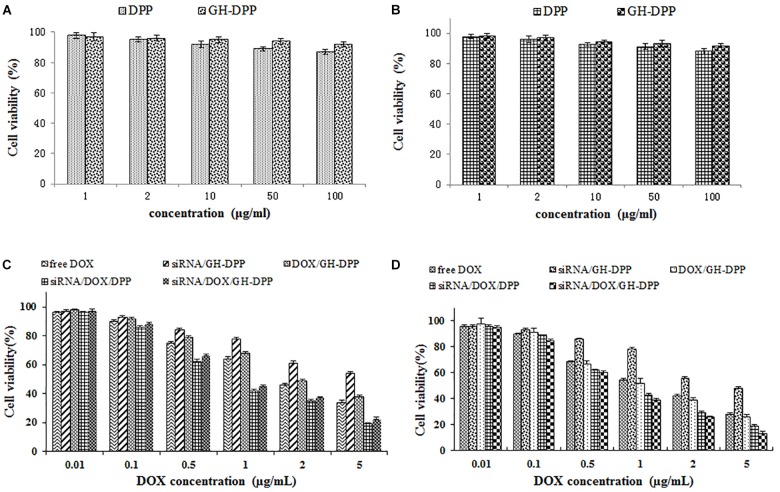
The cell viability of blank nanoparticles against **(A)** A549 cells and **(B)** HepG2 cells for 48 h. The cell viability of drug formulations against **(C)** A549 cells and **(D)** HepG2 cells for 48 h.

The cytotoxicity of blank nano-carriers was determined using the MTT assay. The cytotoxicity of two blank nano-carriers was below 15% at the concentration of 10 to 100 μg/mL (Figure 5A). The results suggested that DPP and GH-DPP nanoparticles could be used in drug delivery materials due to their negligible toxicity.

The viability of A549 and HepG2 cells was evaluated after incubations with free DOX, DOX/GH-DPP, siRNA/GH-DPP, siRNA/DOX/DPP, and siRNA/DOX/GH-DPP nanoparticles for 48 h. Figure 5B showed that all of five drug formulations exhibited similar dose-dependent cytotoxic effects, and that the co-delivery nanoparticles groups (siRNA/DOX/DPP and siRNA/DOX/GH-DPP) showed higher cytotoxicity compared to free drug treatment groups. The half maximal inhibitory concentration (IC50 value) of siRNA/DOX/DPP and siRNA/DOX/GH-DPP nanoparticles against HepG2 cells was measured to be 1.02 and 0.76 DOX μg/mL, respectively, which were lower than that of free DOX (1.86 DOX μg/mL). The results suggested that co-delivery nanoparticles for DOX and Bcl-2 siRNA could enhanced inhibitory effect of DOX. This was due to the fact that sensitivity of HepG2 cells to DOX was enhanced owing to down-regulation of BCL-2 by RNA interference (Cao et al., 2011). As shown in Figures 5C,D, siRNA/DOX/GH-DPP nanoparticles exhibited higher toxicity against HepG2 cells than other DOX formulations, while, it was different at same treatment with A549 cells. The possible explanation was that GA-receptors were over-expressed on HepG2 cells, which enhanced cellular uptake of DOX and siRNA via GA receptor-mediated endocytosis. Whereas, the siRNA/DOX/GH-DPP nanoparticles against A549 cells showed lower cytotoxicity than siRNA/DOX/ DPP nanoparticles. The different cytotoxicity against HepG2 cells and A549 cells might due to different expressed level of GA-receptor on two tumor cells (Tian et al., 2010).

### Cellular Uptake of siRNA/DOX/GH-DPP Nanoparticles and Suppression of BCL-2 Expression

**FIGURE 6:**
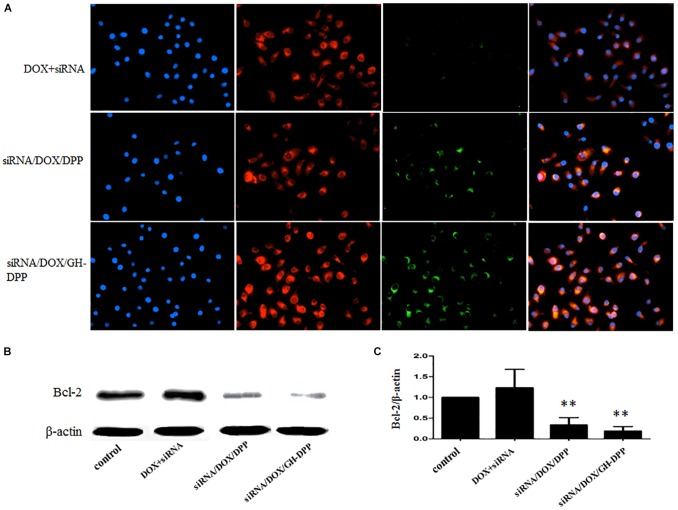
Cellular uptake and western blot analyses. **(A)** The images of HepG2 cells treated with mixture of free DOX and FITC-labeled siRNA, siRNA/DOX/DPP or siRNA/DOX/GH-DPP nanoparticles for 4 h, respectively. FITC channel (green) for FITC-labeled siRNA, TRITC channel (red) for DOX, and DAPI channel (blue) for nucleus were presented simultaneously. **(B,C)** Protein expression of BCL-2 evaluated by western blot analysis with different treatments. ^∗∗^*P* < 0.01 vs. control.

The cellular uptake of siRNA/DOX/GH-DPP nanoparticles was investigated through fluorescence microscope. Green and red fluorescence signals indicate the uptake of siRNA and DOX, respectively, while blue fluorescence signals show the nuclei stained with DAPI. Overlays of three fluorescence picture revealed the distribution of DOX and siRNA in the cytoplasm. As shown in Figure 6A, there were obvious red fluorescence signals in cytoplasm of HepG2 cells incubating with three drug formulations, indicating that DOX was taken up by tumor cells. There was little green fluorescence signals in the group treated by mixture of free DOX and siRNA, indicating that little siRNA were taken up by tumor cells. Compared to drug-loaded DPP nanoparticles, stronger green fluorescence signals were found in HepG2 cells incubating with DOX/GH-DPP nanoparticles. This result was due to the coverage of GA-HA conjugate, which increase the amounts of drugs via GA-receptor-mediated endocytosis. The down-regulation of BCL-2 gene in HepG2 cells was assessed by western blot assays. After treated with siRNA/DOX/DPP and siRNA/DOX/GH-DPP nanoparticles, the expression of BCL-2 protein was inhibited obviously in comparison with the control group (Figures 6B,C), suggesting that the up-regulation of BCL-2 in HepG2 cells could be reversed by RNA interference basing on GH-DPP nanoparticles.

### *In vivo* Biodistribution of GH-DPP Nanoparticles

**FIGURE 7:**
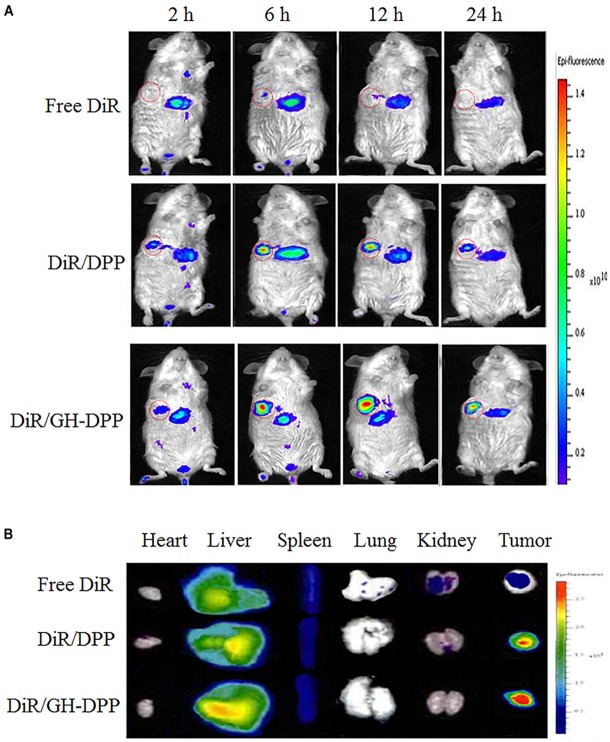
**(A)** Real-time NIRF images of H22 tumors-bearing mice after tail vein injection of free DiR, DiR/DPP and DiR/GH-DPP nanoparticles for 24 h. The tumors are circled in red. **(B)** *Ex vivo* NIRF images of organs and tumors excised at 24 h.

DiR-loaded nanoparticles were prepared to investigate the biodistribution of GH-DPP *in vivo* (Frangioni, 2003). After injection of DiR formulations, fluorescence signals could be monitored in liver and tumor. As shown in Figure 7, there were strong fluorescence signals in the tumor for DiR-loaded nanoparticles compared to free DiR, indicating the nano-carrier could enhance drug accumulation in tumor region (Nichols and Bae, 2014). Moreover, the fluorescence intensity of DiR-loaded GH-DPP nanoparticles in the tumor was greater than that of DiR-loaded DPP nanoparticles. This may be due to the fact that GH-DPP nanoparticles increased accumulation in the liver cancer cells via liver-targeting delivery, and decreased the uptake by normal cells. After injection for 24 h, major organs and tumors were extracted for fluorescent intensity evaluation. Similar to DiR biodistribution in Figure 7A, the DiR-loaded GH-DPP treatment group show strongest fluorescent signals in tumor region (Figure 7B).

### *In vivo* Anti-tumor Effect of siRNA/DOX/GH-DPP Nanoparticles

**FIGURE 8:**
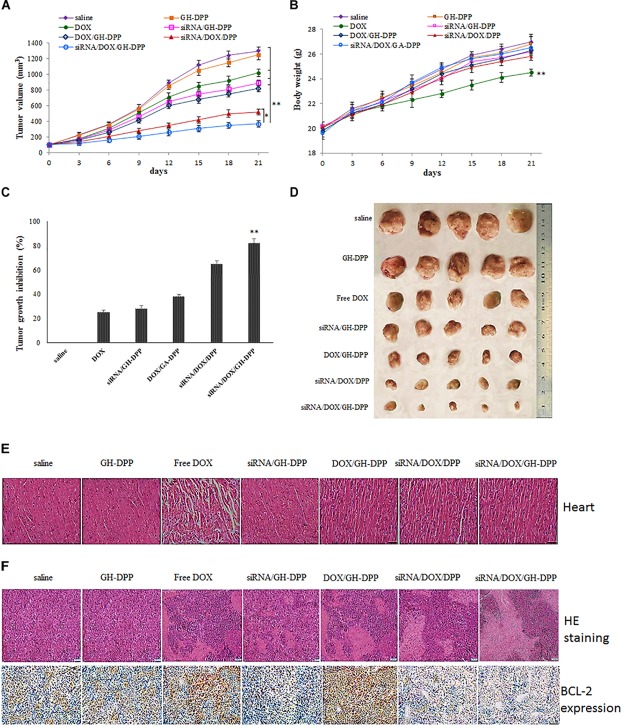
Inhibition of tumor growth by injection of physiological saline (control), blank GH-DPP nanoparticles, free DOX, siRNA/DPP, DOX/DPP, siRNA/DOX/DPP or siRNA/DOX/GH-DPP nanoparticles, respectively. **(A)** Tumor growth curves; **(B)** Body weight changes; **(C)** The tumor growth inhibition rate; **(D)** excised tumors of each group; **(E)** Histological observation of heart for H22 tumor-bearing mice treated with different drug formulations; **(F)** The histological features of H22 subcutaneous tumor sections are characterized by H and E and BCL-2 immunohistochemical analysis. The data represent the mean of the tumor volume or body weight from five mice ± SD; ^∗^*P* < 0.05 and ^∗∗^*P* < 0.01.

The combination of DOX and Bcl-2-siRNA was used in anti-hepatoma therapy. The anti-tumor effect of siRNA/DOX/GH-DPP nanoparticles was evaluated in the H22 tumor-bearing mice. As shown in Figure 8, the groups treated with saline and blank GH-DPP nanoparticles showed a rapid growth in tumor size, and no significant difference was observed between the blank GH-DPP group and the control group, indicating that the GH-DPP nanoparticles was biocompatible. In contrast, the groups treated with drug formulations showed obvious growth inhibition. *In vivo* tumor inhibition ratio (IR) of co-delivery nanoparticles for DOX and Bcl-2 siRNA was higher than GH-PDD nanoparticles for delivery of DOX or siRNA alone, indicating that combined therapy of DOX and Bcl-2 siRNA improved antitumor efficacy. Interestingly, siRNA/DOX/GH-DPP nanoparticles showed stronger anti-tumor effect than siRNA/DOX/DPP nanoparticles. This may be due to GA-HA conjugate promoting the accumulation of drug-loaded nanoparticles in tumor region, resulting in higher anti-hepatoma efficacy than siRNA/DOX/DPP nanoparticles.

Figure 8B showed that the body weight of mice treated with free DOX was lower than those treated with drug-loaded nanoparticles, indicating that GH-DPP nanoparticles decreased the systemic toxicity of DOX. As shown in Figure 8E, obvious intercellular vacuolation and dissolution of myocardial fibers were observed in the group of free DOX, indicating that the injection of free DOX induced significant cardiotoxicity. By contrast, there was no obvious degeneration of myocardial fibers in the groups which were injected by drug-loaded nanoparticles. These results showed that combined therapy basing on nano-carriers improved the anti-tumor effect and alleviated the systemic toxicity of DOX.

The tumors were extracted for H and E staining to evaluate the antitumor effect. As shown in Figure 8F, tumor cells treated with co-delivery system exhibited obvious karyolysis and pyknosis with more cytoplasmic vacuolation in comparison to single drug formulation, indicating that combination therapy exhibited higher antitumor effect. In comparison with siRNA/DOX/DPP nanoparticles, the siRNA/DOX/GH-DPP nanoparticles induced more shrunk nuclei and lower cellular density, suggesting that introduction of GA-HA promote the liver-targeting delivery of drugs, resulting in more effective treatment. The expression of BCL-2 protein was evaluated in the tumor by immunohistochemical assay. The high expression of BCL-2 protein was observed in the groups of free DOX and DOX/GH-DPP nanoparticles. By comparison, the group treated with co-delivery systems showed obvious suppression of BCL-2 expression (Cao et al., 2011).

## Discussion

Liver cancer has become one of the highest incidences of malignant tumor in the world. Conventional chemotherapy has severe system toxicity, and always fails in MDR (Perez-Herrero and Fernandez-Medarde, 2015). Some efforts have been focused on the combination of two or more therapeutic approaches with different mechanisms. The combination of chemotherapy drugs and RNA interference has attracted more attention for the enhanced sensitivity of drugs against tumor cells due to the silence of oncogene (Li et al., 2018). Moreover, nanoparticles for drug delivery have been proven as the useful vehicles of anti-tumor drugs or gene for liver-targeting delivery. The nano-carriers could accumulate in tumor region via active-targeted manner when they are modified by liver-targeting moiety, resulting in loss of side effect from drugs (Chen et al., 2014).

In this study, we prepared the GH-DPP nanoparticles for co-delivery of DOX and Bcl-2 siRNA for liver cancer therapy. The siRNA/DOX/GH-DPP nanoparticles were spherical in shape, negative in zeta potential with an average particle size of 185.4 nm. There was an obvious difference in zeta potential between siRNA/DOX/GH-DPP (negative) and siRNA/DOX/DPP nanoparticles (positive). This was due to the introduction of the negatively charged GA-HA conjugate which induced the shift of surface charge of nano-carriers. The co-delivery system of DOX and Bcl-2 siRNA showed time-dependent sustained release *in vitro*. Compared to DPP nanoparticles, GH-DPP nanoparticles showed slower DOX release. This might due to the fact the coverage layer (GA-HA) delay the release of DOX from GH-DPP.

*In vitro* cytotoxicity test showed that siRNA/DOX/GH-DPP nanoparticles exhibited a better therapeutic effect than delivering DOX or Bcl-2 siRNA alone. This is due to that fact that co-delivery of DOX and Bcl-2 siRNA produce a synergistic anti-tumor effect in which sensitivity of HepG2 cells to DOX was enhanced owing to down-regulation of BCL-2 by RNA interference. Moreover, siRNA/DOX/GH-DPP nanoparticles exhibited higher cytotoxicity than siRNA/DOX/DPP nanoparticles against HepG2 cells (GA-receptor over-expressed). Interestingly, the cytotoxicity of siRNA/DOX/GH-DPP against A549 cells (no GA-receptor) was lower than that of siRNA/DOX/DPP. The possible explanation was that the introduction of GA-HA conjugate promotes the cellular uptake of drug-loaded GH-DPP nanoparticles by HepG2 cells via GA-receptor-mediated endocytosis, leading to higher cytotoxicity (Wu J. et al., 2018). However, there was no GA receptor on A549 cells, and drug-loaded DPP nanoparticles (positive charged) were easily taken up by tumor cells, resulting in higher cytotoxicity than drug-loaded GH-DPP nanoparticles (negative charged).

Figure 6A showed that DOX or siRNA can be effectively taken up by HepG2 cells compared with mixture of free DOX and siRNA. There were stronger fluorescence signals in HepG2 cells incubated with drug-loaded GH-DPP than drug-loaded DPP nanoparticles. This result may be due to the coverage of GA-HA conjugate, which increase the amounts of cellular uptake via GA-receptor-mediated endocytosis (Yan et al., 2018). *In vivo* near-infrared fluorescence imaging shows that the fluorescence intensity of DiR-loaded GH-DPP nanoparticles in the tumor was greater than that of DiR-loaded DPP nanoparticles. This may be due to the fact that DiR-loaded GH-DPP nanoparticles could be accumulated in the tumor tissue by liver-targeting delivery manner (Fan et al., 2015).

As shown in Figure 8, there was no significant difference in body weight and cardiotoxicity between the blank GH-DPP group and the control group. By contrast, the treatment of free DOX induced obvious intercellular vacuolation and dissolution of myocardial fibers, showing significant cardiotoxicity. This result suggested that the GH-DPP nanoparticles were biocompatible and useful for the delivery of chemotherapy drugs (Sun et al., 2018). Compared with nano-formulations for delivery DOX or siRNA alone, siRNA/DOX/GH-DPP nanoparticles showed stronger anti-tumor effect, indicating combination therapy could improve the anti-tumor efficiency by enhancing the sensitivity of cancer cells for chemotherapy drugs through inhibiting the expression of Bcl-2 protein (Chen et al., 2014). Compared to siRNA/DOX/DPP nanoparticles, siRNA/DOX/GH-DPP nanoparticles exhibit stronger antitumor effect. These results showed that the introduction of GA-HA conjugate was helpful to promote the accumulation of drug-loaded nanoparticles in tumor region, resulting in higher anti-hepatoma efficacy (Cai et al., 2016).

## Conclusion

Doxorubicin-loaded DPP nanoparticles were self-assembled and then complexed successively with Bcl-2 siRNA and GA-HA conjugate to prepare a co-delivery system. The GH-DPP nanoparticles could simultaneously deliver siRNA and DOX into HepG2 cells, and GA-receptor-mediated internalization significantly increased the cellular uptake efficiency. *In vitro* and *in vivo* anti-tumor effects revealed that siRNA/DOX/GH-DPP nanoparticles could suppress the expression of Bcl-2 gene, enhanced cell apoptosis, and exhibited higher anti-tumor effect. The results showed that GH-DPP nanoparticles are efficient nano-carrier for co-delivery of siRNA and hydrophobic drug in combined therapy.

## Ethics Statement

Animal studies were conducted according to the Regulation on Experimental Animals of Animal Research Ethics Committee of WeiFang Medical University.

## Author Contributions

JW, GT, and BZ designed the experiments. GT, BZ, and JW prepared the drug-loaded GH-DPP nanoparticles. GT, HJ, ZG, and RP performed *in vitro* anti-tumor analysis. BL, ZG, and RP made the anti-tumor effect *in vivo*. MQ, BZ, RP, and JW wrote the manuscript and contributed to data analyses.

## Conflict of Interest Statement

The authors declare that the research was conducted in the absence of any commercial or financial relationships that could be construed as a potential conflict of interest.
