# Supplementary figures and images for: Liver-Targeted Combination Therapy Basing on Glycyrrhizic Acid-Modified DSPE-PEG-PEI Nanoparticles for Co-delivery of Doxorubicin and Bcl-2 siRNA
Source: Front Pharmacol. 2019 Jan 22;10:4. doi: 10.3389/fphar.2019.00004 (PMC6349772; doi:10.3389/fphar.2019.00004)

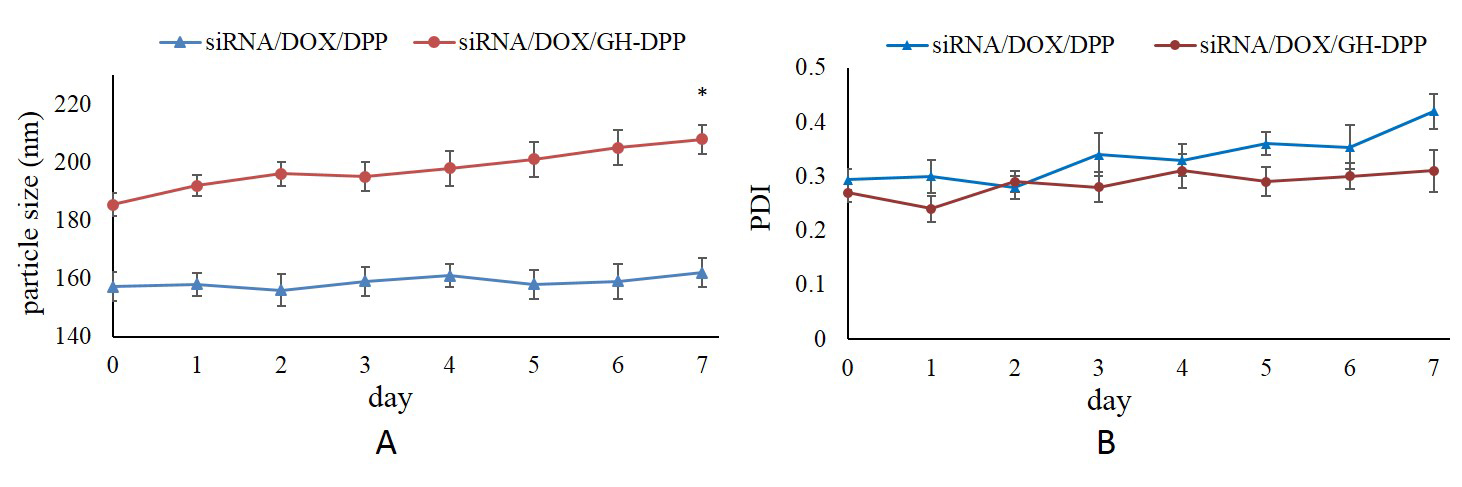

Supplement: Supplementary file 1 [file Image_1.JPEG]
