# Supplementary material for: Liver-Targeted Combination Therapy Basing on Glycyrrhizic Acid-Modified DSPE-PEG-PEI Nanoparticles for Co-delivery of Doxorubicin and Bcl-2 siRNA
Source: Front Pharmacol. 2019 Jan 22;10:4. doi: 10.3389/fphar.2019.00004 (PMC6349772; doi:10.3389/fphar.2019.00004)
Supplement: Supplementary file 2 [file Table_1.DOCX]

**Supplementary Data:**

**Fig. S1.** The stability of siRNA/DOX/DPP and siRNA/DOX/GH-DPP in RPMI 1640 with 10% FBS medium at 37℃ for 7 days. *p <0.05 vs 0 d.
